# A novel quasi-solid state electrolyte with highly effective polysulfide diffusion inhibition for lithium-sulfur batteries

**DOI:** 10.1038/srep25484

**Published:** 2016-05-05

**Authors:** Hai Zhong, Chunhua Wang, Zhibin Xu, Fei Ding, Xinjiang Liu

**Affiliations:** 1 National Key Laboratory of Science and Technology on Power Source, Tianjin Institute of Power Source, Tianjin, 300384, P. R. China

## Abstract

Polymer solid state electrolytes are actively sought for their potential application in energy storage devices, particularly lithium metal rechargeable batteries. Herein, we report a polymer with high concentration salts as a quasi-solid state electrolyte used for lithium**-**sulfur cells, which shows an ionic conductivity of 1.6 mS cm^−1^ at room temperature. The cycling performance of Li-S battery with this electrolyte shows a long cycle life (300 cycles) and high coulombic efficiency (>98%), without any consuming additives in the electrolyte. Moreover, it also shows a remarkably decreased self-discharge (only 0.2%) after storage for two weeks at room temperature. The reason can be attributed to that the electrolyte can suppress polysulfide anions diffusion, due to the high ratio oxygen atoms with negative charges which induce an electrical repulsion to the polysulfide anions, and their relatively long chains which can provide additional steric hindrance. Thus, the polysulfide anions can be located around carbon particles, which result in remarkably improved overall electrochemical performance, and also the electrolyte have a function of suppress the formation of lithium dendrites on the lithium anode surface.

Sulfur is a promising cathode material with a high theoretical specific capacity of 1672 mAh g^−1^. When paired with a lithium metal anode to form rechargeable lithium-sulfur (Li-S) batteries, the theoretical specific energy density can reach as high as 2600 Wh kg^−1^, making it an attractive chemical power source to meet increased demand from portable electronic devices, transportation and large-scale stationary energy storage applications. In addition, sulfur has the advantages of abundance, low cost, and non-toxicity.

Although Li-S battery have been proposed for more than 30 years, implementation of this rechargeable battery technology is still hindered by some critical obstacles, including the loss of active sulfur material, low coulombic efficiency, high self-discharge rate, and short life^1^. These problems mainly stem from the dissolution and diffusion of the intermediate lithium polysulfide (Li_2_S_x_, 3 ≤ × ≤ 8) in the nonaqueous electrolyte^2^, especially the diffusion issues. During charge-discharge of the cell, the dissolved lithium polysulfides diffuse from the cathode to the lithium anode through the separator and induce a so-called “shuttle effect”. Such shuttle effect causes the loss of the active sulfur materials, lowers the coulombic efficiency and also consumes the lithium metal anode^3^.

To overcome these problems, many strategies have been successfully developed, including novel cathode materials (carbon-sulfur^4^,^5^,^6^,^7^ and polymer coated sulfur composites^8^,^9^,^10^), electrode engineering methods^11^,^12^,^13^, functional polymer binders^14^,^15^,^16^, as well as electrolytes additives^17^,^18^,^19^. Despite the promising progress, the use of organic-electrolyte soaked porous separator was not able to fully address the polysulfide diffusion issue. Moreover, the charging and discharging processes of Li-S batteries involve not only polysulfide anions but also polysulfide anionic radicals^20^,^21^, both of which are highly reactive and possibly react with electrolyte solvents and polymer binder. From the viewpoint of chemical stability, the most suitable solvent and binder for Li-S batteries are ether-based solvents and polymers. However, they also fail to achieve perfect energy storage performance. The ether-based solvent, such as 1,3-dioxolane (DOL), has a good solubility of sulfur and lithium polysulfides, which can improve the reaction kinetics. However, these polysulfide intermediates in these solvent will diffuse to anode. The ether-based polymer, such as poly(ethylene oxide) (PEO) as polymer electrolyte matrix, shows a low ion conductivity at room temperature, and also can dissolve lithium polysulfide at higher temperature. According to the above, liquid electrolyte show a high ion conductivity but cause polysulfides diffusion issues, solid electrolyte can inhibit polysulfides diffusion but is hardly used at room temperature. The quasi-solid state electrolyte (QSSE) is superior to a liquid electrolyte in term of preventing dissolution of organic cathode compounds, as reported by Honma^22^, and it is also superior to solid state electrolytes in term of ion conductivity. Therefore, modification of the conventional separator by QSSE has been considered to be more practical for the performance improvement of Li-S batteries.

Herein, we report a new polymer-in-salt electrolyte (the “PIS electrolyte”) as quasi-solid state electrolyte (QSSE) and its application in Li-S batteries. It is demonstrated that PIS electrolyte can prevent the diffusion of polysulfide anions by special polymer structures, which result in a perfect long cycle life and high coulombic efficiency. In addition, it also shows a decreased self-discharge after two week storage.

## Results

**Figure 1:**
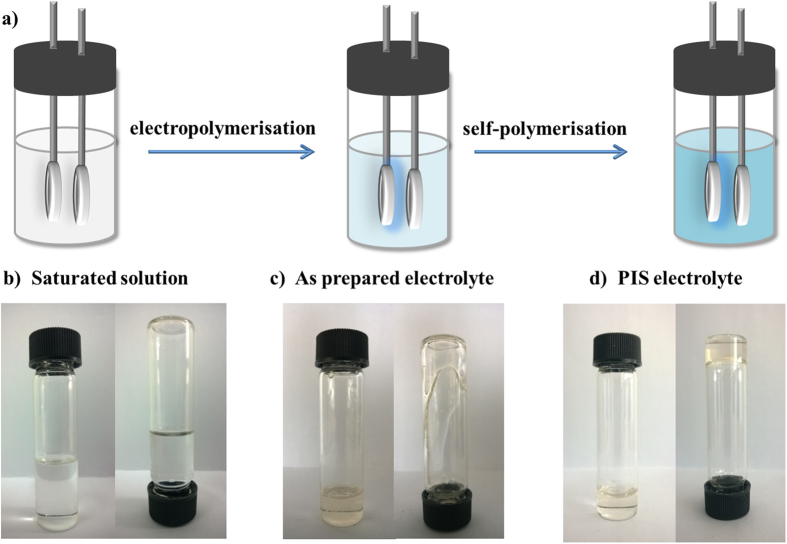
(**a**) Schematic of the procedure for fabricating PIS electrolyte, (**b**) LiTFSI/DOL saturation electrolyte, (**c**) As-prepared electrolyte after electropolymerisation and treatment by saturation electrolyte, (**d**) PIS electrolyte after further self-polymerisation of as-prepared electrolyte for two weeks at room-temperature.

Electro-polymerization method was adopted to synthesize the PIS electrolyte, because it doesn’t need any catalysts for polymerization, and the product can be used directly without further purification. LiTFSI was chosen as the lithium salt for saturation, and DOL monomer is the solvent for electropolymerisation. The polymer matrix after polymerisation has a high O atom ratio, and the O atoms as well as polysulfide anions possess electro-negativity. Since they may have a mutual intermolecular repulsive force, and the polymer exert a strong steric hindrance effect on the polysulfide anions, the PIS electrolyte would prevent polysulfide anion diffusion and avoid the shuttle effect issues. Figure 1a shows the procedure of fabricating PIS electrolyte: after electro-induced polymerisation of the saturation solution and further self-polymerisation for two weeks, the PIS electrolyte was obtained. Figure 1b–d are typical photographs of the electrolyte with different forms which corresponding to the prepared steps. It can be seen that the saturation electrolyte (LiTFSI/DOL) kept the characteristics of the liquid with its low viscosity (Fig. 1b). After electro-induced polymerisation for several hours, the viscosity of the as-prepared electrolyte increased significantly compared to that of the saturation electrolyte, but it could slowly flow down along the bottle wall when being inverted (Fig. 1c). Further self-polymerisation of as-prepared electrolyte for more than two weeks at room-temperature produced a transparent solid polymer electrolyte which was immobile upon inversion of its container (Fig. 1d).

**Figure 2:**
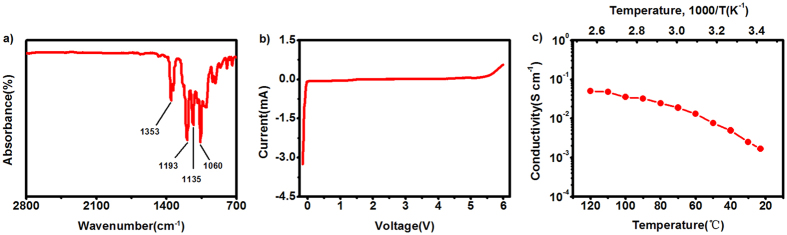
(**a**) FT-IR spectra of PIS electrolyte, (**b**) Line scans voltammetry of PIS electrolyte (Li/PIS/stainless steel) with a scan rate of 0.5 mV s^−1^ at 25 °C, (**c**) Temperature dependency of the ionic conductivities of PIS electrolyte (Li/PIS/stainless steel) over the temperature range 23 °C to 120 °C.

Figure 2a shows the FT-IR spectra of PIS electrolyte. Strong absorptions at 1060 cm^−1^, 1135 cm^−1^ and 1193 cm^−1^ are attributed to the stretching vibration of C-O bond of the polymer matrix, and the absorptions at 1353 cm^−1^ are attributed to the stretching vibration of C-F bond of LiTFSI^23^. The Polymer molecular weight of PIS electrolyte determianted by gel permeation chromatography (GPC) was summarizaed in Table S1. The PIS have a greatly higher molecular weight than DOL, indicating it can provide a steric hindrance to polysulfides diffusion. In view of the practical application, the electrochemical stability of the PIS electrolytes should be first considered for ensuring a reliable performance in rechargeable lithium batteries. Therefore, line scan voltammetry (LSV) was carried out to investigate the electrochemical voltage window of the PIS electrolyte, and the data were obtained by the Li/PIS/stainless steel cell at a scan rate of 0.5 mV s^−1^ at 25 °C (Fig. 2b). It was shown that the electrolyte remains stable at up to 5.5 V in positive scan, and also it was stable at the lower voltage to 0 V (*vs.* Li/Li^+^), which was acceptable for most electrode materials for rechargeable lithium batteries. The oxidation potential of PIS electrolyte is much higher than LiTFSI in conventional carbonate based solvents. In order to explain this phenomenon, a contrast LSV experiment was carried out and the results was shown in Figure S1: the electrolyte 1 M LiTFSI EC:DMC (1:1, v/v) with stainless steel cathode could remain stable at up to 5.1 V, while the electrolyte with aluminium foil cathode shows abruptly increased anodic current at about 4.0 V. This result is also similar to previous reports^24^, which proves that the stainless steel is more stable than aluminium foil with LiTFSI salts in liquid electrolyte. Because aluminium foil as current collector will cause a pitting corrosion at potentials above 3.7 V *vs.* Li/Li^+ ^25. Figure 2c shows the ionic conductivity of *in situ* polymerized PIS electrolyte uptaken by Celgard membranes as a function of temperature, which was determined by the methods reported in previous studies^26^,^27^. As shown, the PIS electrolyte had a conductivity of 1.67 × 10^−3^ S cm^−1^ at 23 °C.

**Figure 3:**
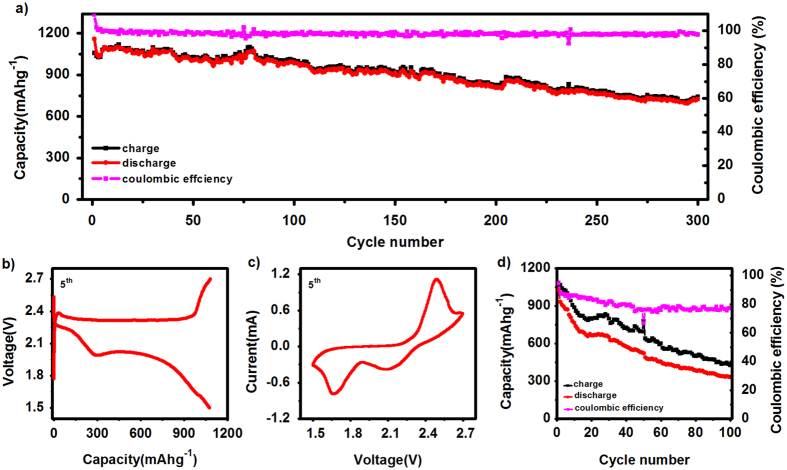
(**a**) Charge/discharge capacity and coulombic efficiency vs. Number of cycles of the PIS electrolyte in Li-S batteries with a current 100 mA g^−1^ between 1.5–2.7 V at room temperature, (**b**) The 5^th^ cycle voltage-capacity profile of Li-S cell with PIS electrolyte, (**c**) The 5^th^ cyclic voltammetry of Li-S cell with PIS electrolyte at a scan rate 0.2 mV s^−1^, (**d**) The cycle performance of sulfur cathode with LiTFSI/TEGDME saturation solution electrolyte at a current 100 mA g^−1^.

To demonstrate the unique advantages of PIS electrolytes, we further exploit its application in rechargeable Li-S batteries. A Li-S battery using sulfur/carbon composite as cathode active materials (details see Figure S2) and PIS as electrolyte exhibited good electrochemical performance (Fig. 3). The cycling performance of Li-S cell with PIS electrolytes is shown in Fig. 3a, which was tested at a current of 100 mA g^−1^ within a potential range of 1.5–2.7 V at room temperature. An initial discharge capacity of 1160 mAh g^−1^ and a charge capacity of 1058 mAh g^−1^ were attained, corresponding to a coulombic efficiency of more than 100%, due to the irreversibility of the side reaction. Thereafter, the coin cell gave high reversible discharge/charge capacities in the following cycles with efficiency above 98%, which was superior to previous reports of similar systems^28^,^29^. The Li-S battery with PIS electrolyte showed excellent cycling life over 300 cycles, retaining a specific capacity of 720 mAh g^−1^. The capacities of the cells fluctuated because the temperature also fluctuated slightly during the long testing process. It should be noted that the high performance of Li-S cell was achieved by the PIS electrolyte applied, without the addition of any consuming agents such as LiNO_3_^30^, LiI^31^, P_2_S_5_^32^ or polysulfide^33^ additives to improve coulombic efficiency and cycling stability.

A typical charge-discharge profile (5^th^ cycle) of the Li-S cell with PIS electrolyte is shown in Fig. 3b. It is showed that the mean value of its discharge potential was 2.0 V. The curve showed two discharge plateaus at around 2.1 and 1.9 V with the PIS electrolyte, this represented the conversions of S_8_ to Li_2_S_4_ and Li_2_S_4_ to Li_2_S, respectively. There was a sloping line at the end of the discharge, suggesting that Li_2_S was formed, which is similar to that with ether-based liquid electrolytes reported elsewhere^34^. Slight polarisation was observed compared to the cells with typical DOL-DME liquid electrolyte (Figure S3), because of the high viscosity of the PIS electrolyte. Figure 3c shows the 5^th^ cyclic voltammograms of the Li-S cell with PIS electrolyte at a scan rate of 0.2 mV s^−1^: two reduction peaks and one oxidation peak is observed with obvious polarisation^35^, since the high viscosity may not favour ionic transportation. We also show the cycling results of Li-S cell using LiTFSI/TEGDME saturation solution as electrolyte (Fig. 3d). It shown a capacity of 1026 mAhg^−1^ and efficiency of 94.5% at the first cycle, but the cycling performance is really poor. The capacity and efficiency are only 330.9 mAhg^−1^ and 77.5% after 100 cycles, respectively. TEGDME and the polymer matrix of PIS electrolyte have similar structure, but the cycles performance have an obvious difference, due to the polymer matrix have a higher O atom ratio and longer chains. The high performance of Li-S cell was mainly attributed to that the PIS electrolyte prevented polysulfide anion diffusion and avoided the shuttle effect issues.

**Figure 4:**
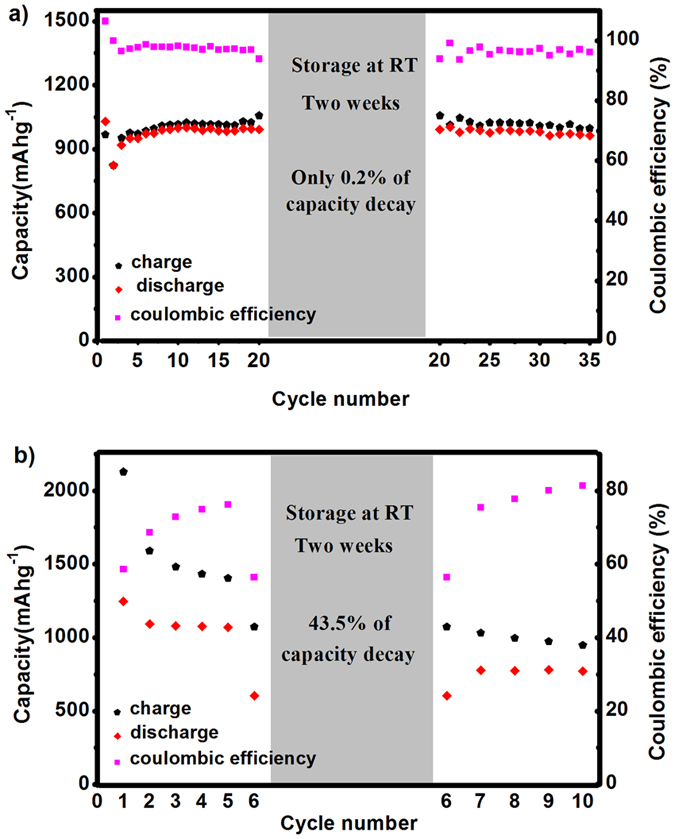
Charge/discharge capacity and coulombic efficiency vs. number of cycles of Li-S cell with (**a**) PIS electrolyte and (**b**) 1 M LiTFSI DOL/DME (v/v = 1:1) electrolyte before (1^st^–19^th^) and after (21^st^–35^th^) a storage time of two weeks.

Low self-discharge is another criterion used to judge the practicality of energy-storage devices. Unfortunately, Li-S batteries have strong self-discharge tendencies, as do nickel-cadmium or traditional nickel-metal hydride batteries. Since the dissolution of polysulfide anions is inevitable in Li-S batteries when using non-aqueous electrolytes, after charging, sulfur/high-order polysulfide anions continue to slowly dissolve in the electrolyte even in a resting state^1^. When batteries are rested, self-discharge occurs because the active material gradually dissolves and migrates to the anode due to the concentration gradient and then reacts with lithium metal followed by conversion into high-order polysulfide anions, resulting in a decrease of open circuit voltage and discharge capacity. In order to investigate the self-discharge behaviours of Li-S cell with PIS electrolyte, we studied it by storing it for two weeks at room temperature (Fig. 4). As shown in Fig. 4a, the Li-S cell with PIS electrolyte before storage (1^st^–19^th^ cycle) shows stable cycling performance. Then the cell was cut-off during the discharge process after the first discharge plateau representing of the conversion of S_8_ to Li_2_S_4_. After cell storage at room temperature for two weeks, the cell was re-cycled under identical conditions. It showed a similar cyclic performance (21^st^–35^th^) as that before storage (1^st^–19^th^). There was no obvious change between the discharge capacities at the 19^th^ and 20^th^ cycles (995 and 993 mAh g^−1^, respectively). The small difference arose from the charge capacity of the 20^th^ cycle undergoing a slight increase, so the coulombic efficiency decreased from 97% (19^th^) to 94% (20^th^), but this was recovered in the remaining cycles. When the discharge capacity reached 310 mAh g^−1^ at 20^th^ cycle, the cell was cut-off, this was according to the first discharge curve (Figure S4), which is shown the first discharge step (S_8_ to Li_2_S_4_) of initial cycles ended at this point. The results of reference Li-S cell with 1 M LiTFSI DOL/DME is shown in Fig. 4b. Because the cycle performance of reference cell is poor at a low current of 100 mAg^−1^, we just cycled it for only five cycles, then storage it for two weeks. The self-discharge behaviour of the reference cell with two drops of electrolyte (1 M LiTFSI DOL/DME, v/v = 1:1) shows discharge capacities of 1071.4 mAh g^−1^ and 605.7 mAh g^−1^, before and after being stored at room temperature. The results suggest that the PIS electrolyte can remarkably mitigate the self-discharge, with only 0.2% capacity reduction compared to 43.5% for the reference cell. The reasons may be attributed to the effect that PIS electrolyte can restrict lithium polysulfides in small and localised regions.

## Discussion

**Figure 5:**
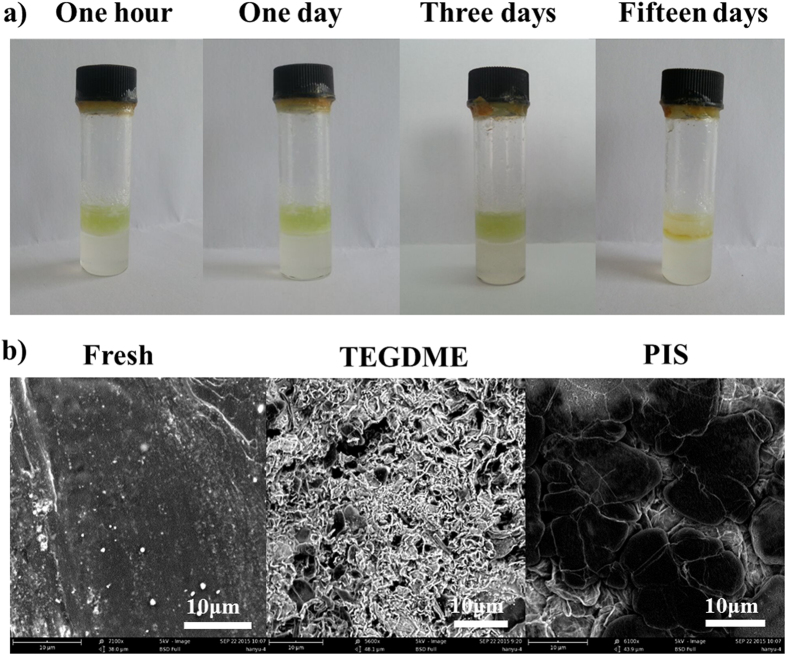
(**a**) Lithium polysulfide diffusion experiments, the upper layer is lithium polysulfide solution with DME solvent, the lower layer is PIS electrolyte layer, (**b**) Typical scanning electron microscopy images of metallic lithium anodes after long cycling experiments in Li-S cells. “Fresh” is lithium metal without any treatment, “TEGDME” is lithium metal with LiTFSI/TEGDME saturation electrolyte after 100 cycles, “PIS” is Lithium metal with PIS electrolyte after 300 cycles.

The results are also supported by the lithium polysulfide diffusion and lithium metal stability experiments (Fig. 5). As shown in Fig. 5a, the upper layer with light yellow solution was lithium polysulfide solution in DME, the lower layer was PIS electrolyte. It is seen from this figure, the polysulfide anions have not diffused into the PIS electrolyte layer even after standing for 15 days. Due to the effect of PIS electrolyte, it is difficult for the polysulfide diffuse to the lithium metal anode surface, and thus the perfect results were achieved with long cycling stability and high coulombic efficiency. The typical photograph of metallic lithium anode with PIS electrolyte is get by disassembling the Li-S cell after 300 cycles (Figure S5). It is shown that no yellow colour species can be observed on the surface of lithium anode. Figure 5b show the typical scanning electron microscopy images (SEM) of metallic lithium anodes after long-term cycling in Li-S cells. From these images, lithium anodes with both TEGDME and PIS electrolyte had an obvious change compared with fresh lithium, but the PIS electrolyte shows much slighter damage level of metallic lithium anode compared with the TEGDME. The morphology of metallic lithium anodes demonstrates that the PIS electrolyte system can effectively inhibit the polysulfide diffusion, thus it can reduce the corrosion. And also the ultrahigh lithium salt concentration and high viscosity can suppress the formation of lithium dendrites^36^, and decrease the solubility of lithium polysulfide due to the common ion effect^37^.

The PIS electrolytes not only suppress polysulfide, but also prolong the stable time of lithium metals under atmosphere (around 65% humidity), as shown in Figure S6. From this figures, we can see that the small region of lithium metal with traditional electrolyte become black after 2 min, indicating that lithium have reacted with the water vapour in the air. The regions become larger after 5 min, but others doesn’t show any change. Until 30 min later, the edge of lithium metal with as prepared electrolyte and PIS electrolyte start to reacted, which indicate the protecting effect of PIS electrolyte is better than as prepared electrolyte. The results also suggest that PIS electrolyte have a function of enhancing the stability of lithium metal.

In summary, a new polymer in salt was proposed as a quasi-solid state electrolyte for the next-generation of high-energy Li-S batteries, which was prepared by electrochemically triggered LiTFSI/DOL saturated solution polymerisation. For application in Li-S batteries, it was demonstrated that PIS electrolytes can not only inhibit the diffusion of polysulfide anions, but also protect lithium metal anode. Thus, the Li-S cell with PIS electrolyte exhibit an excellent cycle performance with high coulombic efficiency (>98%), and with long cycle stability which still released a capacity of 720 mAh g^−1^ after 300 cycles, especially without any consuming additives. PIS electrolyte also shows a strong mitigation of self-discharge at room temperature, with only 0.2% capacity reduction after two week storage. It was expected that the PIS electrolyte would offer a new and important approach to improve the cyclic stability and mitigate the self-discharge of Li-S batteries.

## Methods

### Synthesis

The PIS electrolyte is prepared as follows: firstly, excess lithium bis(trifluoromethane sulphonyl)imide (LiN(SO_2_CF_3_)_2_, LiTFSI) (Guotai Huarong, China) was added to purified DOL (Super dry, J & K Seal) solvent to produce a saturated solution. Then, the electro-polymerisation of a low molecular weight polymer was carried out in a sealed glass cell, which had a stainless steel disc (diameter, 20 mm) as its working electrode and lithium foil as the counter and reference electrode. The working electrode was polished and cleaned before use. The electropolymerisation of the DOL monomer saturated solution (5 ml) was performed under potentiostatic control at OCV-4.5 V *vs.* Li/Li^+^ with a current of 0.1 mA, which was then followed by a constant voltage charge step (24 hours, 4.5 V *vs.* Li/Li^+^). Finally, an electrolyte with high viscosity, was obtained which was designated the “as prepared” electrolyte. The *in situ* polymerisation PIS electrolyte uptakes by the celgard membranes, which was caused by immersing the celgard membrane in the “as-prepared” electrolyte, and then further self-polymerising for two weeks in the sealed bottle at room-temperature (around 25 °C), the electrolyte without fluxility which marked it as PIS electrolyte. The sulfur/carbon composite was prepared by melt-diffusion method. According to Ji *et al.* report^38^, Ketjen-carbon and sulfur were ground together (3:7, m/m) and sealed in a glass tube, then heated at 155 °C for 8 h. The characterisations of this composite are given in Supplementary Figure S1.

### Characterisations

The FT-IR spectra of PIS electrolyte was recorded on AVATAR 360 spectrometer (Nicolet Instrument Corp., USA) in range of 700–2800 cm^−1^. The average molecular weight (M_n_ and M_w_) of the prepared polymer of PIS electrolyte were determined by gel permeation chromatography (GPC) using an Agilent 1100 GPC equipped with a PLgel 10 μm MIXED-B column and an RI detector. X-ray diffraction (XRD) analysis was conducted with an XRD-6000 diffractometer (Shimadzu, Japan), using Cu *K*_*α1*_ radiation and a scan rate of 3° min^−1^. The morphology was observed on a Quanta 200 scanning electron microscope (SEM, FEI Company, The Netherlands) at a dry room (0.1% RH). Thermo-gravimetric (TG) analysis was carried out on a TG-DTA6300 thermal analyser (PE Company, USA) at a heating rate of 15 °C min^−1^ in N_2_ flow.

### Electrochemistry

The electrochemical stability of the PIS electrolyte was evaluated by linear sweep voltammetry in blocking-type cells using a stainless steel working electrode, a lithium foil counter electrode, and the PIS electrolyte uptake by celgard membranes as the electrolyte and separator at a scan rate of 0.5 mV s^−1^ at room temperature with a electrochemical working station. The electrode was fabricated by mixing S/C (7:3) composite, Super P, poly(vinylidene difluoride) in weight ratio of 8:1:1. The slurry was cast on an Al current collector and dried overnight at 50 °C in vacuum, typical electrode S loading is 1.5 mg cm^−2^. The coin cells (CR2032) were assembled with the electrode, and lithium foil as counter electrode and PIS electrolyte uptake by celgard separator in an argon-filled glove box. The bare cells were assembled for comparison, with two drops of 1 M LiTFSI DOL:DME (1:1 by volume) electrolyte. The discharge and charge measurements were carried out on a Land Battery Test System (Wuhan, China) at room temperature. The ionic conductivity of the PIS electrolyte (*σ*) was measured in blocking-type cells, which were fabricated by sandwiching PIS electrolyte with celgard separator, between a lithium foil electrode and a stainless steel electrode (Li/PIS/stainless steel). Impedance data were obtained with a Princeton Applied Research Solartron Analytical over the frequency range 0.1 Hz to 100 kHz and over a temperature range of 23 °C to 120 °C in a thermostatically-controlled container. The ionic conductivity of the prepared PIS electrolyte was calculated by *σ* = *d*/(*S* × *R*_*b*_), where *σ* is the ionic conductivity, *R*_*b*_ is the bulk resistance, *d* is the thickness of the PIS electrolyte, and *S* is the area of the electrode.

## Additional Information

**How to cite this article**: Zhong, H. *et al.* A novel quasi-solid state electrolyte with highly effective polysulfide diffusion inhibitation for lithium-sulfur batteries. *Sci. Rep.* **6**, 25484; doi: 10.1038/srep25484 (2016).

## Supplementary Material

Supporting Information
