# Supplementary material for: A novel quasi-solid state electrolyte with highly effective polysulfide diffusion inhibition for lithium-sulfur batteries
Source: Sci Rep. 2016 May 5;6:25484. doi: 10.1038/srep25484 (PMC4857097; doi:10.1038/srep25484)

Supporting information

**A novel quasi-solid state electrolyte with highly effective polysulfide diffusion** **inhibitation for lithium-sulfur batteries**

Hai Zhong, Chunhua Wang, Zhibin Xu, Fei Ding*, Xinjiang Liu

Figure S1 Line scans voltammetry of 1 M LiTFSI EC:DMC (1:1, v/v) electrolyte with stainless steel and aluminium foil as working electrode at scan rate of 0.5 mV s^-1^.


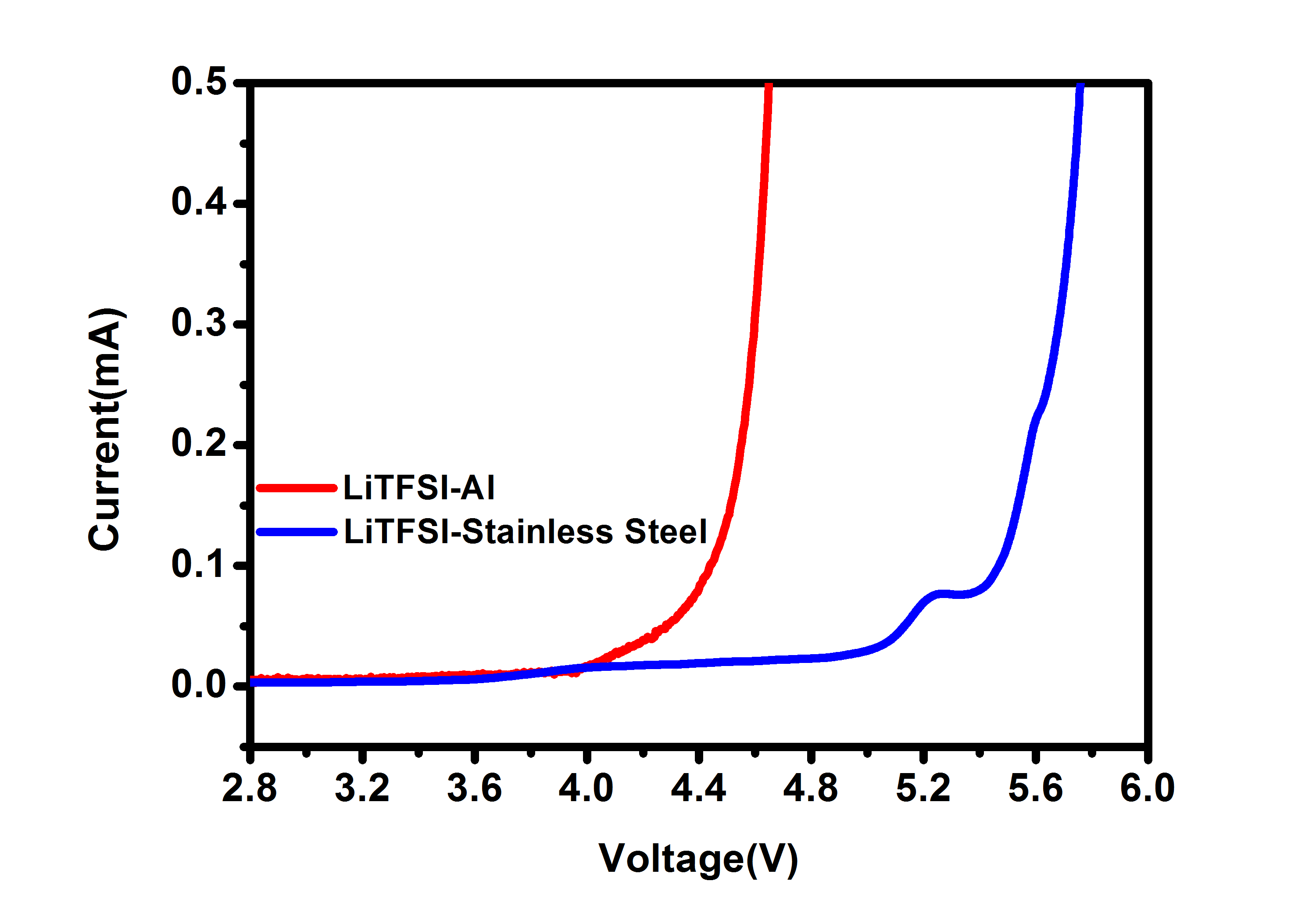


Figure S2 (a) The XRD pattern of sulfur/KJ-carbon (S/C) composite treated by melt-diffusion strategy, KJ-carbon and sulfur, (b) TG curves of S/C composite recorded in N_2_ with a heating rate of 15 °C min^−1^, (c) SEM image of S/C composite, (d) SEM image of KJ-carbon.


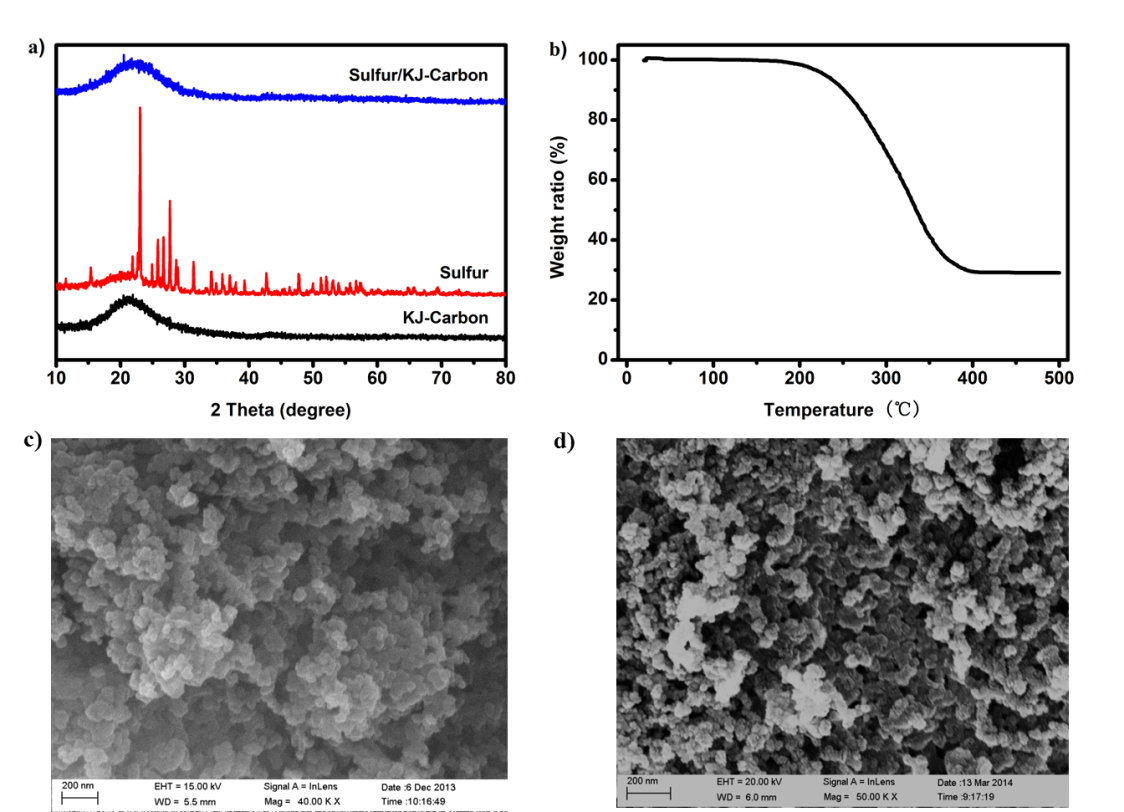


Table S1 The molecular weight test results.

| Polymer |  | M_n_ |  | M_w_ |  | M_w_/M_n_ |
| --- | --- | --- | --- | --- | --- | --- |
|  |  | 23,489 |  | 52,363 |  | 2.229 |

Figure S3 The 5^th^ cycle charge/discharge curves of the Li-S cell with its PIS electrolyte and reference cell (1 M LiTFSI DOL/DME).


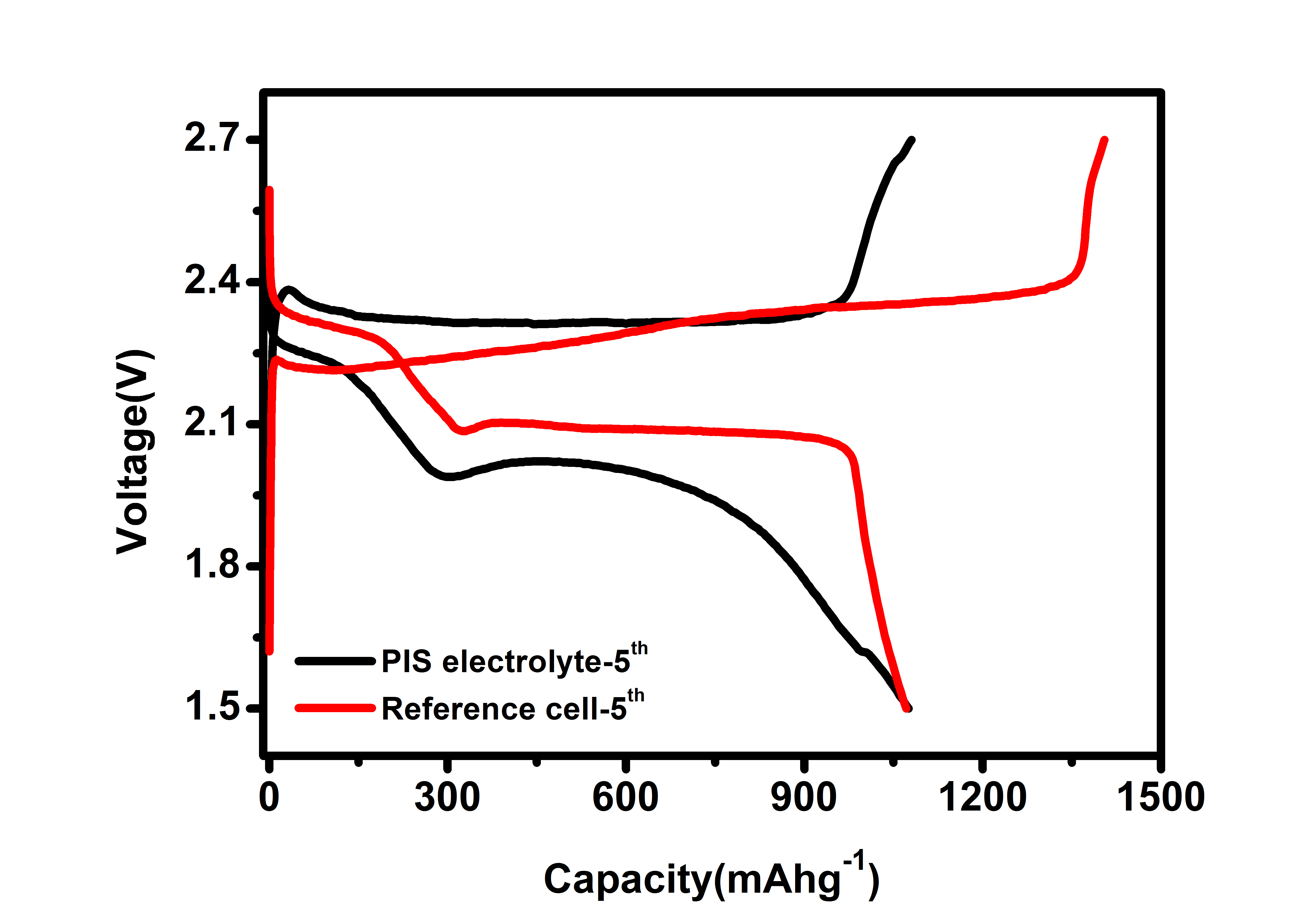


Figure S4 The 1^st^ and 20^th^ cycle discharge curves of the Li-S cell with its PIS electrolyte.


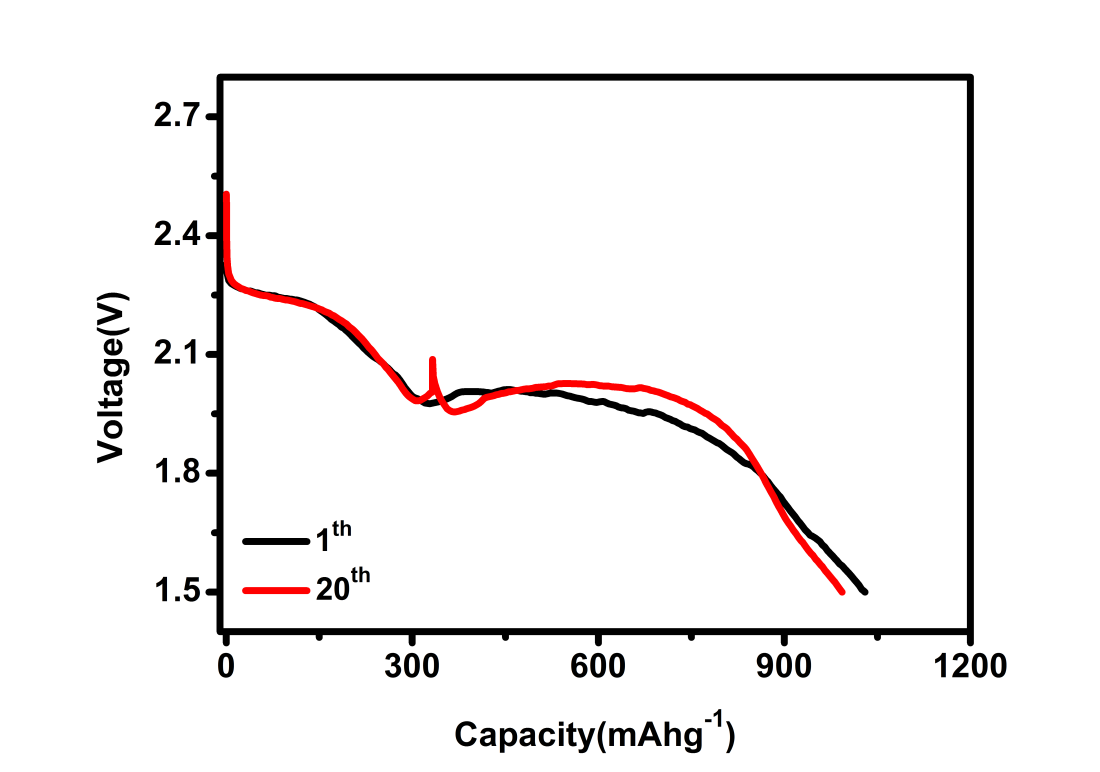


Figure S5 The typical photograph of metallic lithium anode disassemble from Li-S cell after 300 cycles.


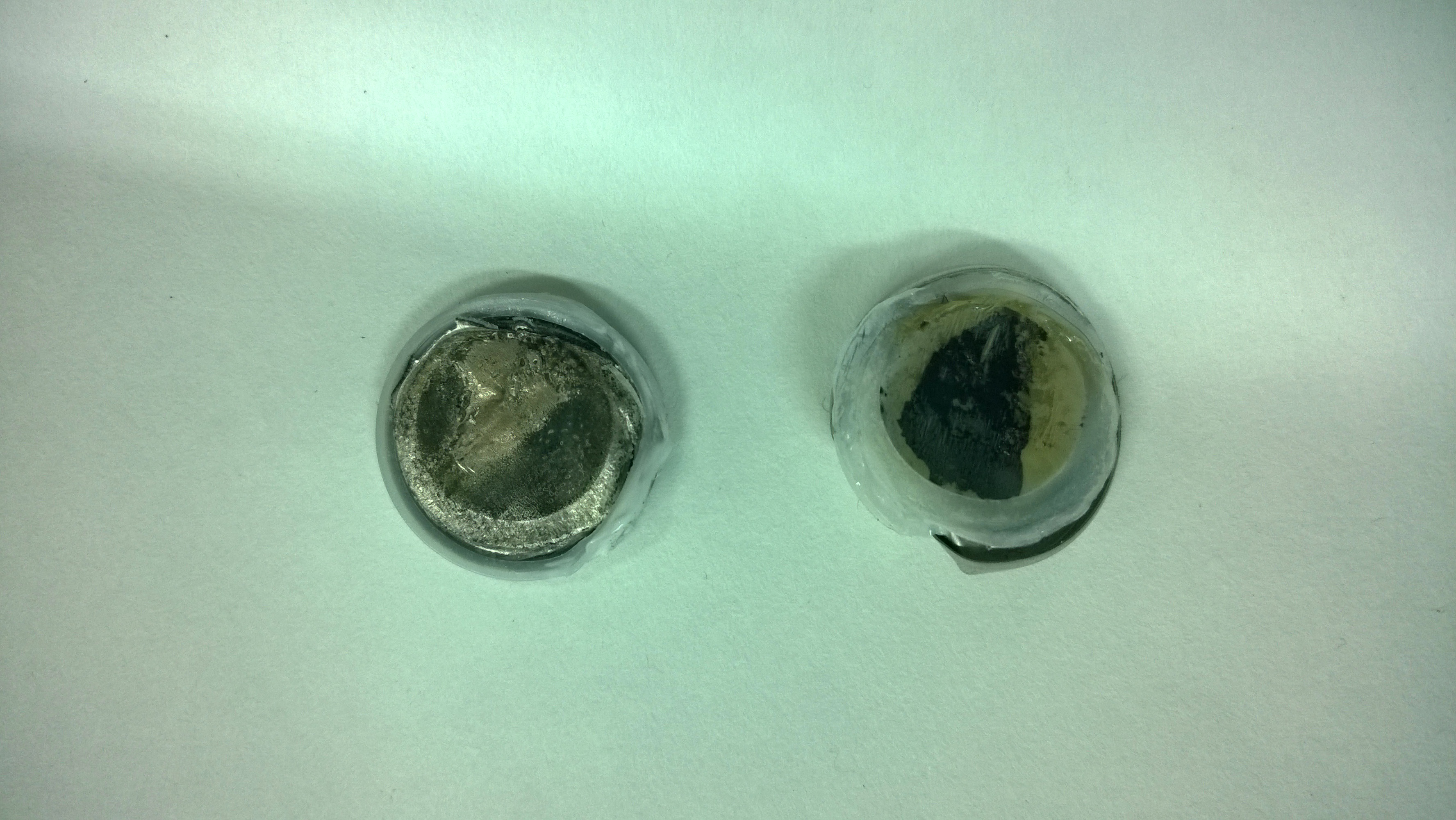


Figure S6 The stability of lithium metals under atmosphere.


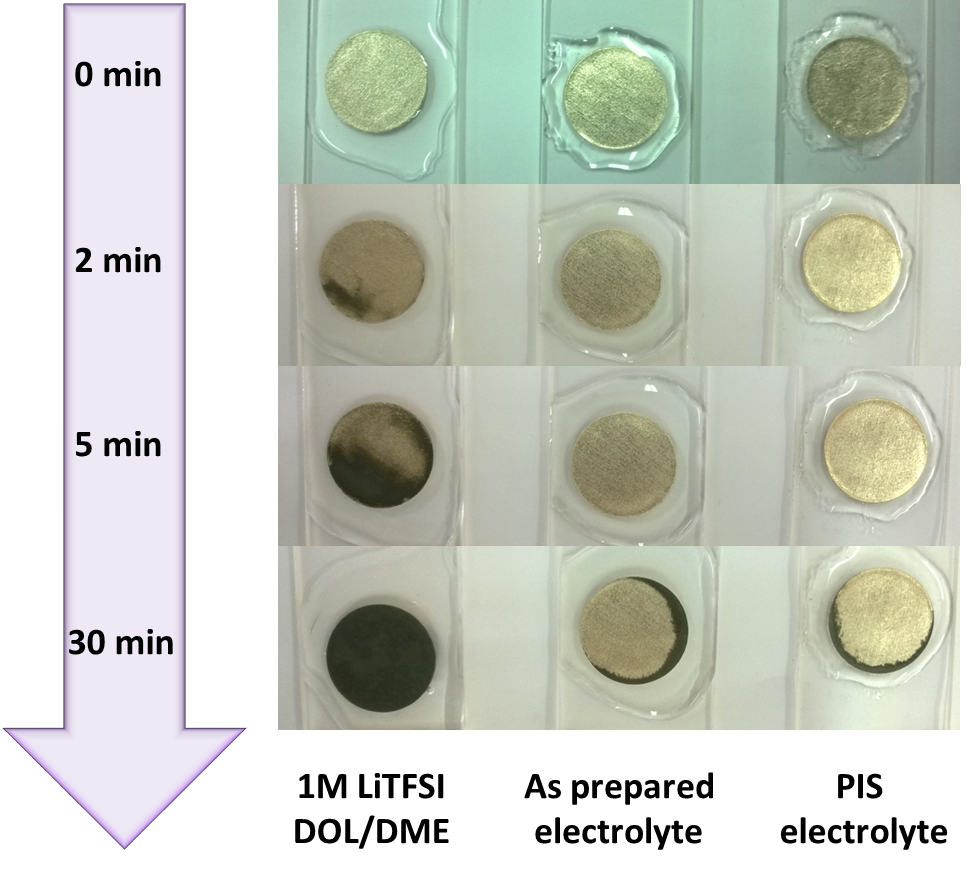

Supplement: Supporting Information [file srep25484-s1.doc]
